# Life Threatening Severe QTc Prolongation in Patient with Systemic Lupus Erythematosus due to Hydroxychloroquine

**DOI:** 10.1155/2016/4626279

**Published:** 2016-07-12

**Authors:** John P. O'Laughlin, Parag H. Mehta, Brian C. Wong

**Affiliations:** ^1^New York Methodist Hospital, Department of Internal Medicine, 506 6th Street, Brooklyn, NY 11215, USA; ^2^New York Methodist Hospital, Department of Internal Medicine, Division of Cardiology, 506 6th Street, Brooklyn, NY 11215, USA

## Abstract

We present a case of a syncopal episode resulting from significant QT interval prolongation in a patient on hydroxychloroquine for the treatment of systemic lupus erythematosus and end stage renal disease. The patient had been treated with hydroxychloroquine for two years prior to presentation. After thorough workup for secondary causes of QT interval prolongation hydroxychloroquine was discontinued and the patient's QT interval shortened. The patient was treated with mexiletine to prevent sudden ventricular arrhythmias, which was unique compared to other documented cases in which lidocaine was used. The patient was noted to have mild prolongation of the QT interval on electrocardiogram prior to initiation of hydroxychloroquine therapy which was exacerbated by its use and may have been caused due to toxicity from underlying renal failure.

## 1. Introduction

QT interval prolongation is the result of abnormal repolarization of the ventricular myocardium resulting in lengthening of the QT interval on electrocardiogram [1–3]. In females the normal corrected QT interval is 470 ms, with males slightly lower at 450 ms [4]. It can be the result of congenital genetic mutations in cardiac myocyte ion channels [5], acquired from electrolyte derangements (hypocalcemia, hypokalemia, and hypomagnesemia), or from use of numerous medications. Common classes of medications include antibiotics, antiarrhythmics, antidepressants, and antipsychotics (Table 1) [6]. Clinical manifestations of prolonged QT interval include syncope and sudden cardiac death from a fatal cardiac arrhythmia known as Torsades de Pointes (TdP). TdP is a form of polymorphic ventricular tachycardia with a heart rate greater than 100 beats per minute with characteristic twisting around the isoelectric baseline every 5–20 beats [7, 8].

Of the documented medications known to cause QT interval prolongation hydroxychloroquine (HCQ) is extremely rare in the literature. HCQ is an antimalarial that has become the cornerstone for treatment of systemic lupus erythematosus (SLE) [9]. HCQ has a long half-life ranging from 32 to 50 days with a modest side effect profile with most common reactions associated with the gastrointestinal system [10] and ocular toxicity [11]. The drug is commonly utilized in treatment of SLE for patients with end stage renal disease (ESRD); however the exact dosing and toxicity are not well established and need further study [12]. The common cardiac toxicities of HCQ are not well defined. Chen et al. 2006 reported QT interval prolongation with associated TdP in a patient on chronic HCQ treatment [13], and Morgan et al. 2013 described QT interval prolongation in a patient with SLE while taking HCQ [14]. We present a unique case of severe QT interval prolongation in a patient with SLE and ESRD on chronic HCQ therapy which lead to a life threatening complication of syncope and resultant head trauma.

## 2. Case

**Figure 1:**
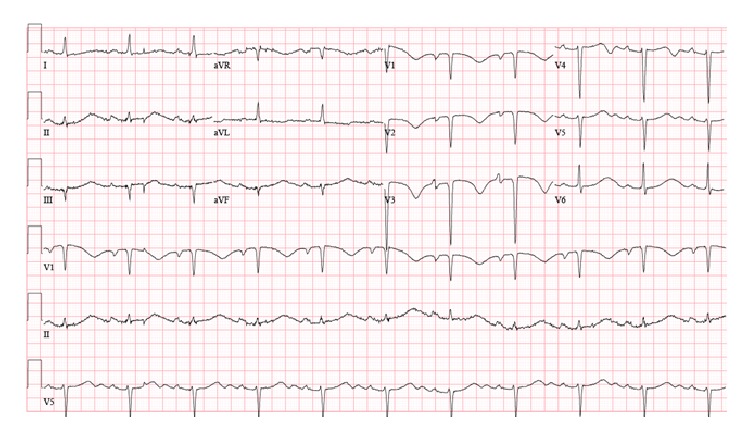
Admission ECG: 1st-degree heart block, with QTc 689 ms.

**Figure 2:**
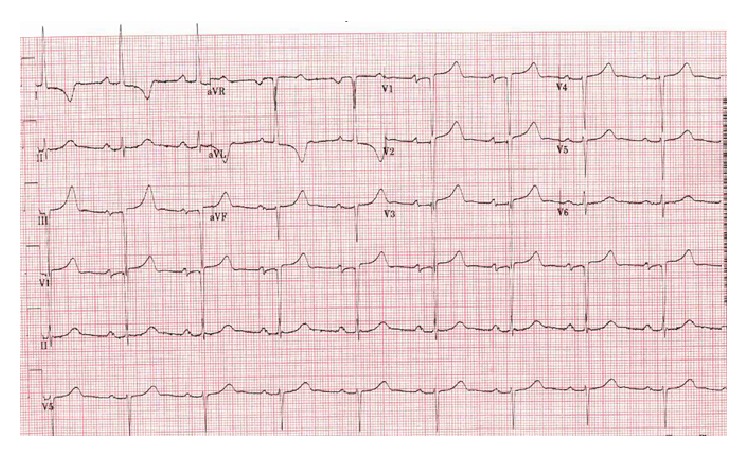
Two years prior to admission. QTc 500 ms. Patient's baseline ECG.

A 50-year-old female with a past medical history of SLE diagnosed 20 years ago, ESRD on hemodialysis and atrial fibrillation on anticoagulation, presented to the emergency department after a syncopal episode. The patient was sitting on the side of her bed when she suddenly lost consciousness falling to the floor striking the right side of her face. She sustained significant facial trauma; however there were no signs of intracranial hemorrhage on CT scan of the brain. She described palpitations with chest pressure prior to the event. Initial electrocardiogram (ECG) reported by emergency medical services did not reveal any acute arrhythmias, such as TdP. Due to the complaints of chest pressure she was evaluated for cardiac ischemia utilizing cardiac biomarkers, CKMB and Troponin I. A total of three sets were completed every eight hours apart; all studies were reported within normal limits ruling out cardiac ischemia. Troponin I values were less than 0.045 ng/mL and CKMB values were all less than 0.5 ng/mL. ECG on presentation revealed 1st-degree AV block with corrected QT (QTc) interval of 689 ms (Figure 1). Patient's ECG from two years prior showed a baseline prolonged QTc interval of 500 (Figure 2). She was evaluated for reversible causes of QT interval prolongation including electrolyte derangements, medications, and SLE flare. Her electrolytes were within normal limits including potassium (3.9 mmol/L), magnesium (2.3 mg/dL), phosphorous (4.2 mg/dL), and calcium (9.0 mg/dL). C3 levels were low, with normal C4 and ESR, and anti-dsDNA was negative making SLE flare unlikely. Patient continued to have dialysis on her normally scheduled days.

**Figure 3:**
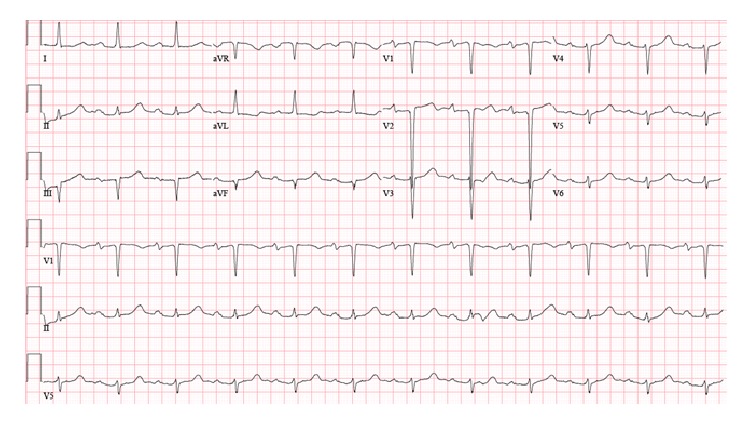
One week into admission after discontinuation of hydroxychloroquine and treatment with mexiletine 150 mg BID. QTc 518 ms. Shortening of QTc to near patient's baseline of 500 ms.

After evaluation by electrophysiology there was concern for presumed TdP due to her clinical presentation and grossly abnormal QTc interval. She was placed on mexiletine 150 mg BID and was initially recommended to have defibrillator implantation to protect against sudden cardiac death from cardiac arrhythmias. Evaluation of the patient's medications revealed that she was taking HCQ for the past two years for treatment of refractory SLE. HCQ was discontinued and the patient's QT interval slowly decreased to a QTc interval of 500 over the next 7 days (Figure 3). She was monitored on the telemetry unit for one week and did not have any further arrhythmic events. After further discussion with the patient's Cardiologist and Rheumatologist she had been noted to have a prolonged QTc interval of approximately 500 ms while on therapy with HCQ. She was trialed off the medication for two months without resolution of the QT interval abnormality, possibly due to undiagnosed underlying SLE cardiomyopathy. The patient's ejection fraction on echocardiography one year prior was estimated at 65% with Doppler parameters consistent with abnormal relaxation suggesting heart failure with preserved ejection fraction. Implantable defibrillator placement was discussed with the patient prior to admission due to the prolonged baseline QTc interval. However, she was reluctant to proceed as she did not want indwelling devices due to concern for possible Permacath placement in the future for dialysis if her arteriovenous fistula malfunctioned.

The risks and benefits of continuing HCQ were discussed with the patient, and she decided she wanted to continue the medication due to well controlled SLE flares. She had noted adverse reactions to steroid therapy and limited treatment options for SLE due to her underlying renal failure. We discussed the risks of recurrent syncopal episodes if she was to continue HCQ therapy, and the high risk for intracranial bleed while on anticoagulation if she was to have another syncopal episode. Final recommendations were made to proceed with defibrillator placement if she was to continue with HCQ to protect against life threatening arrhythmias. She had a subcutaneous defibrillator placement and was to be restarted on HCQ as an outpatient with dose reduction and close monitoring of QTc interval.

At three-month follow-up the patient remained off HCQ as after discussion with her Rheumatologist the risks related to syncope and possible intracranial bleed while on anticoagulation treatment greatly outweighed the benefits of HCQ therapy. She continues to be closely monitored for SLE flares, and treatment decisions are to be strategized as clinical issues arise. Subcutaneous implantable defibrillator interrogation showed no recorded events at three months. Her ECG continues to show a prolonged QTc interval of 508 ms. Due to the prolonged QTc interval she is still at high risk of ventricular arrhythmias despite discontinuation of HCQ. Therefore, the subcutaneous implantable defibrillator is providing secondary prevention of sudden cardiac death due to prolonged QT interval.

## 3. Discussion

QT interval prolongation is of consequential clinical importance as patients are at high risk of traumatic injury from syncopal events or sudden cardiac death from life threatening arrhythmias such as TdP [6, 7]. Without early identification of possible reversible causes of QT interval prolongation patients may have fatal outcomes. The identifiable causes relating to electrolyte derangements, medications, or cardiac ischemia must be quickly rectified. If patients continue to have QT interval prolongation despite optimal medical management patients may need immediate protection from arrhythmias with the use of antiarrhythmic medications such as lidocaine or mexiletine and long term protection with an external or implantable defibrillator.

The decrease in QT interval observed in this patient is likely multifactorial due to discontinuation of the offending agent HCQ and the use of mexiletine. Mexiletine is a class IB antiarrhythmic that acts by blocking sodium channels shortening the plateau phase of the myocardial action potential thereby hastening repolarization rates with resultant shortening of QT interval duration [15].

Of the known medications to cause QT interval prolongation, HCQ is not commonly implicated. With HCQ as a first-line therapy for treatment of SLE, it is important that QT interval be frequently monitored in patients on chronic therapy. HCQ has a significantly long half-life, 32–50 days; however metabolism of the drug is not well understood [10, 11]. Treatment recommendations for SLE include HCQ 400 mg orally once or twice daily with therapy ranging from weeks to months [16]. If prolonged therapy is needed doses should range from 200 to 400 mg daily [16]. The use of HCQ in patients with renal disease is not well documented, and thus patients with renal impairment should be closely monitored while on the drug.

The proposed mechanism by which HCQ causes QT interval prolongation is not well understood. Recently demonstrated in guinea pig sinoatrial node myocytes by Capel et al. 2015, they observed findings consistent with inhibitory effects of HCQ on the hyperpolarization activated current ion channels (also known as “funny current” channels, *I*_f_), along with delayed rectifier potassium currents (*I*_Kr_), and L-type calcium ion currents (*I*_CaL_) [17]. These inhibitory effects on the pacemaker cells were shown to cause delayed rates in depolarization leading to decreased heart rates in the cell line studied [17]. These findings may correlate with a proposed mechanism by which refractory action potentials in cardiac myocytes may lead to prolongation of QT interval due to delayed depolarization and repolarization from abnormal ion currents.

This case is a unique presentation of a patient on HCQ therapy for approximately 2 years, with maintenance dosing at 200 mg oral daily. Due to the renal impairment, medication toxicity may have resulted in the severe QT interval prolongation with resultant cardiac arrhythmia and subsequent syncopal event. A recent retrospective review of patients (*n* = 1,537) on HCQ therapy found no significant correlation between HCQ use and QT interval prolongation [18]. Reported cases of QT interval prolongation with HCQ use are likely related to toxic blood levels due to overdose or abnormal metabolism in patients with liver or renal dysfunction. Therefore, patients with renal and hepatic impairment should consider alternative treatment modalities as they are at highest risk for QT interval prolongation and subsequent arrhythmias. Further investigation into the mechanism of action of HCQ, its metabolism, and other possible cardiac toxicities needs to be further elucidated.

## 4. Conclusion

HCQ has proven to be successful in the treatment of SLE, rheumatoid arthritis, and malaria [19, 20]. The therapeutic benefits are well established; however treatment in specific populations needs to be further investigated to establish more specific recommendations on dosages and contraindicated populations to prevent possible fatal events. With the documented cases of HCQ use and related QT interval prolongation, all patients irrespective of underlying comorbidities should be frequently monitored with electrocardiograms.

## Figures and Tables

**Table 1:** Few of the major classes of medications known to cause QT interval prolongation [6].

Class	Medications
Antibiotics	Quinolone: levofloxacin and moxifloxacin Macrolide: erythromycin and clarithromycin

Antiarrhythmic drugs	Class Ia: quinidine, procainamide, disopyramide Class III: dofetilide, ibutilide, sotalol

Antidepressants	Amitriptyline, desipramine, imipramine, maprotiline, fluoxetine

Antipsychotic	Neuroleptic: haloperidol, droperidol, thioridazine, chlorpromazine Atypical antipsychotics: ziprasidone, risperidone, citalopram

## Abbreviations

SLE: Systemic lupus erythematosus
HCQ: Hydroxychloroquine
QTc: Corrected QT interval
ECG: Electrocardiogram.
